# Differentiation of *H. pylori*-negative and positive gastric cancer via regulatory network analysis 

**Published:** 2020

**Authors:** Saeid Abdi, Mona Zamanian Azodi, Mostafa Rezaei-Tavirani, Mohammadreza Razzaghi, Mohammah Hossein Heidari, Alireza Akbarzadeh Baghban

**Affiliations:** 1 *Gastroenterology and Liver Diseases Research Center, Research Institute for Gastroenterology and Liver Diseases, Shahid Beheshti University of Medical Sciences, Tehran, Iran *; 2 *Proteomics Research Center, Shahid Beheshti University of Medical Sciences, Tehran, Iran*; 3 *Proteomics Research Center, Faculty of Paramedical Sciences, Shahid Beheshti University of Medical Sciences, Tehran, Iran*; 4 *Laser Application in Medical Sciences Research Center, Shahid Beheshti University of Medical Sciences, Tehran, Iran.*; 5 *Proteomics Research Center, School of Rehabilitation, Shahid Beheshti University of Medical Sciences, Tehran, Iran *

**Keywords:** Helicobacter pylori, Gastric cancer, MicroRNA, Regulatory network, Hub

## Abstract

**Aim:**

To understand the molecular difference between *H.pylori* negative and positive gastric cancer, a regulatory network analysis is investigated.

**Background:**

Helicobacter pylori as the one of the most leading causes of gastric cancer is yet to be studied in terms of its molecular pathogenicity.

**Methods:**

Cytoscape version of 3.7.2 with its applications was employed to conduct this study via corresponding algorithms.

**Results:**

A total of 161 microRNAs were identified differentially expressed in the comparison of two groups of gastric cancer including negative and positive with *H.pylori* infection. CluePedia explored the regulatory network and found down-regulation dominant while considering the linked hub genes.

**Conclusion:**

It can be concluded that the presented microRNAs and target genes could have associations with *H.pylori* carcinogenesis in gastric cancer through dysregulation of some vital biological processes. These microRNAs and target genes include hsa-miR-943, hsa-miR-935, hsa-miR-367, hsa-miR-363, hsa-miR-25, and hsa-miR-196b and ADRA1A, KCNA4, SOD1, and SESN3, respectively. However, verification analysis in this regard is required to establish these relationships.

## Introduction

 Different mechanisms are discussed and suggested for *H.pylori* carcinogenesis (1). One of the most known cancers related to *H.pylori* infection, is adenocarcinoma of stomach. It is estimated that approximately above half of the population of the world carries *H.pylori* infection (2). Gastric cancer, while reduced in prevalence, requires more studies since it is accounted as the second cancer-related cause of death in the world (3). The risk of gastric cancer can be reduced by eliminating *H.pylori* (4). Molecular basis analysis should be established to better understand its underlying mechanisms. There are some studies in this regard introducing some proteins, genes, and microRNAs for *H.pylori* infection. It has been identified that the cag pathogenicity island (cag PAI) is linked with increasing risk of gastric cancer in *H.pylori* compared with the strains lacking this site (5). Proteomics as one of the novel molecular approaches assigned Annexin A4 for gastric cancer with *H.pylori* infection (6). Recombinant Antibody Microarray analysis also showed that there is fundamental discrimination in expressed proteins of infection and tumor (7). On the other hand, another valuable field of study in this regard is the evaluation of differentially expressed microRNAs (DEMs) across gastric cancer without *H.pylori* versus infection with *H.pylori* through microarray analysis (8). This approach can detect biomarkers for different kinds of diseases (9). MicroRNAs as one of the most famous pathogenesis of different kinds of cancers, are types that do not code any proteins though they have roles is modulating genes (9). Further, using bioinformatics, additional information especially analysis of regulatory networks could be reached to find biomarker signatures (10). MicroRNAs interacting with hub genes indicate their prominent site in the regulation and may play an essential role in *H.pylori* infection mechanism. Thus, in the study, MicroRNAs analysis in a regulatory network is aimed to gain more insight into *H.pylori* pathogenicity. 

## Methods

The GSE54397 dataset, with GPL15159 platform, was queried for microRNA expression profile analysis between two groups of H.pylori negative (8 controls) and H.pylori positive (8 cases) in gastric cancer. Chang H et.al in 2014 presented a study entitled "microRNA expressions in gastric cancer" and published their research as “Different microRNA expression levels in gastric cancer depending on Helicobacter pylori infection” in 2015 (8). The patients participated in this study were diagnosed with adenocarcinoma of stomach and sampling was carried out from these 16 patients at Seoul National University Bundang Hospital (8). They were demographically matched in terms of gender, age, and H.pylori infection condition. The dataset was retrieved from Gene Expression Omnibus (GEO) database and analyzed through GEO2R. This online software is available on (http://www.ncbi.nlm.nih.gov/geo/geo2r/). At first, to analyze whether the expression profile of these samples is comparable in terms of statistical concept, box plotting was performed and eventually the rest of the analysis was continued. Median-centered samples indicate continuation of the analysis. 

Based on P≤0.01 and log fold-change > 2 threshold in this study, a list of DEmiRNAs was achieved. The GEO2R ranks expression data according to the statistical significance for the top 250 ones. To represent the expression data of the microRNAs across the groups of study, Heatmapper was applied as an online software (http://heatmapper.ca/expression/) (11). For this purpose, the GSE54397 series matrix data file was downloaded and processed for this analysis. Finally, prediction of target genes of DEMs was handled through regulatory network analysis via CluePedia, Cytoscape 3.7.2 app (12, 13). The miRanda source (miRNA-V5-2012-07-19.txt.gz) was the database source for this analysis via CluePedia panel. The kappa cut off score for the interaction between hubs and miRNAs was set to or miRanda-SCORE-v5≥ 0.6. A total of 10 hub genes were assigned. Thereafter, the 10 hub genes from the regulatory network were selected for enrichment analysis by ClueGO+CluePedia to gain more knowledge about their role and contribution in biological processes of genes (14). Kappa statistics was employed to explore the term relationships regarding biological processes. The kappa score grouping was set to 0.5 for this analysis. Other statistical criteria were minimum number of genes per term contribution and percentage of genes per term involvement which were set to 1 and 1, respectively. The p-value correction method in this regard was Bonferroni step down. 

## Results

**Figure 1:**
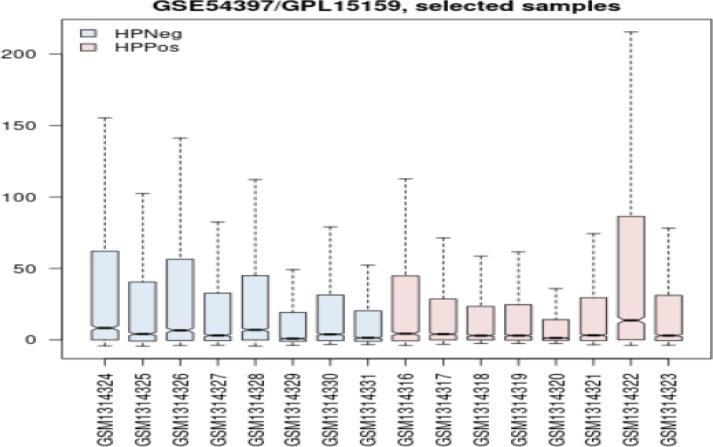
Value distribution comparison across two groups of *H.pylori* negative (blue) and H.pylori positive (pink) for gastric cancer tissue. The distributions are median-centered

In order to evaluate the groups of gastric cancer samples (HP^- ^and HP^+^) in terms of expression comparison, box plot analysis was performed through GEO2R analysis as observed in Figure 1. 

In Figure 1, each group consists of 8 samples whose value distribution indicates their statistical acceptability for further analysis. This fact is inferred from the median-centered nature of samples of comparison. 

Heatmapper (which is an online analyzer) was applied to plot heatmap. Expression amounts of microRNAs in the *H.pylori* negative and *H.pylori* positive samples are presented in the heatmap plot. 

**Figure 2:**
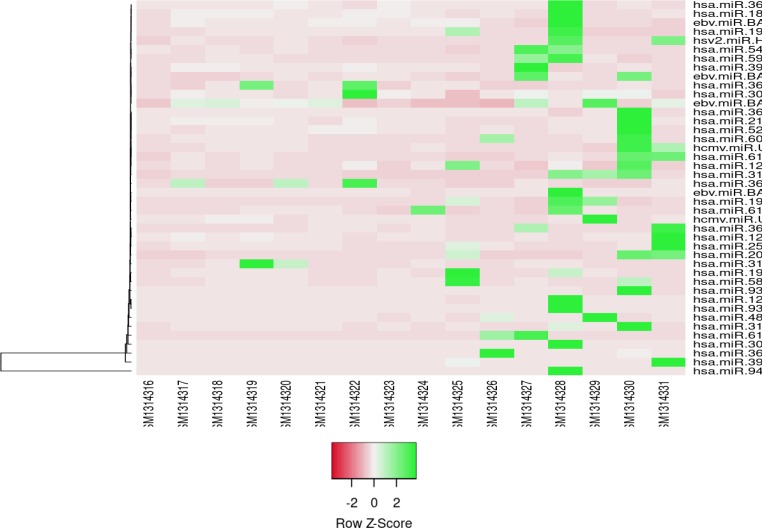
Heatmap presentation of differentially expressed (DE) microRNAs profiles of gastric cancer HP+ and gastric cancer HP- samples. GSM314316-23 and GSM314324-31 refer to gastric cancer HP+ and gastric cancer HP- samples, respectively. Clustering method; average linkage and distance measurement method; Euclidean was applied

**Table 1:** The list of hub miRNAs of regulatory network and their relationship with hub genes

Row	miRNA_ID	Regulation condition	Degree	Hub genes
1	hsa-miR-943	Down	5	ADRA1A,HDAC8,SSX3,TDRKH,ZNF597
2	hsa-miR-935	Down	5	ADRA1A,HDAC8,PLIN2,SOD1,ZNF597
3	hsa-miR-367	Down	5	KCNA4,SESN3,SOD1,SSX3,TESK1
4	hsa-miR-363	Down	5	ADRA1A,KCNA4,SESN3,SOD1,TESK1
5	hsa-miR-25	Down	5	KCNA4,SESN3,SOD1,SSX3,TESK1
6	hsa-miR-196b	Down	5	HDAC8,KCNA4,PLIN2,SESN3,TESK1

**Figure 3:**
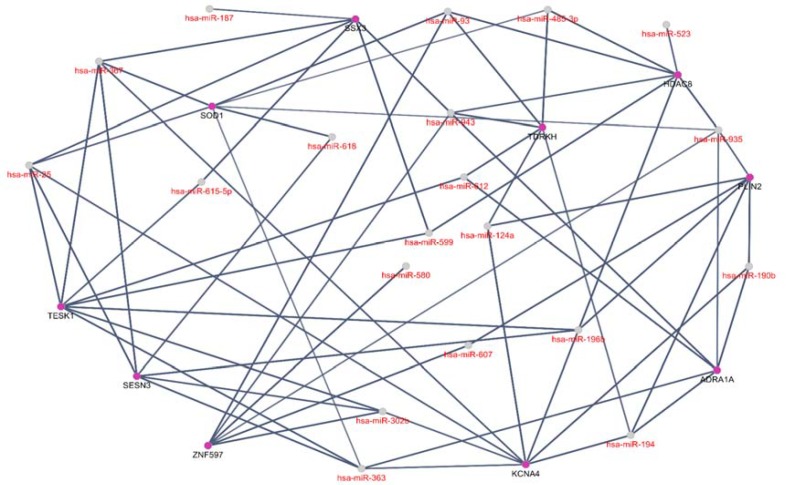
MicroRNA-target network of DE microRNAs and their first neighbor hub genes. A total of 30 nodes and 66 links are presented in the network. The genes are shown with purple color. The miRNA score is considered 0.6

The color of the cells code different values (max-average-min) (see Figure 2).

As indicated in Figure 2, most of the microRNAs are down-regulated and only hsa-miR-3655, hsa-miR-3145-3p, hsa-miR-30c-1, and hsa-miR-367 show up-regulation in the sample with *H.pylori* infection. 

Among the 250 first ranked microRNAs based on significance, 42 significant DE MicroRNAs based on P-Value ≤ 0.01 were selected for further analysis. Among these selected molecules, there were 4 up-regulated and 38 down-regulated DEMicroRNAs. 

The query of up-regulated and down-regulated miRNAs identified 40 elements via CluePedia platform of Cytoscape v.3.7.2. After addition of 10 hub genes, 30 nodes (10 genes and 20 microRNAs) remained as a regulatory network with the connection of 66 links as observed in Figure 3.

The regulatory network in Figure 3 indicates that our DE miRNAs have interactions with some hub genes that could be important in *H.pylori* function in gastric cancer. 

**Figure 4:**
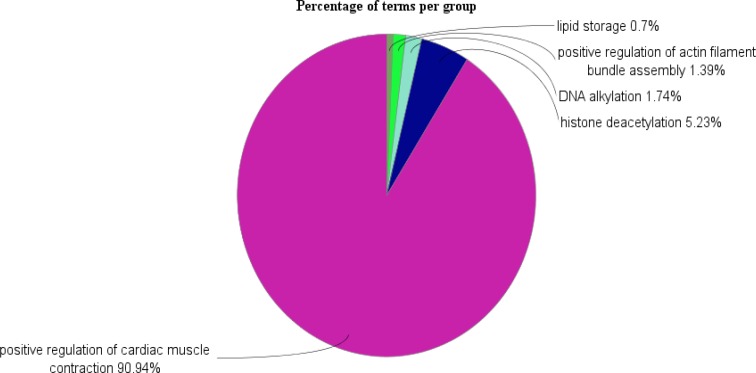
Pie chart view of 10 hub genes of the regulatory network of gastric cancer *H.pylori*-positive enriched in five groups of biological processes

As indicated in Table 1, down-regulation is dominant in the regulatory network. Six DE miRNAs have the highest degree level of regulation in the regulatory network. The degree value above 5 was considered. In Figure 4, positive regulation of cardiac muscle contraction, histone deacetylation, DNA alkylation, positive regulation of actin filament, and lipid storage are introduced as the biological process connected to the hub genes. 

## Discussion

H.pylori is known as one of the famous risk factors for gastric cancer (15). However, there are some cases of gastric cancer without H.pylori infections. To understand the role of this Gram-negative bacterium in gastric cancer development, bioinformatics analysis of DE miRNAs (obtained from microarray analysis in GEO) was handled in this study. Specifically, the relationships between potential miRNAs and genes associated with them via regulatory network could be studied via Cytoscape network analyzer. In this network, dysregulated miRNAs are in connection with genes that whose identification could introduce more knowledge about H.pylori’s contribution to gastric cancer. As inferred in Heatmap display of microRNA expression, the trend of expression in H.pylori infected group is dominantly down-regulation. In addition, the regulatory network constructed by CluePedia also is a down-regulated network with six hub microRNAs. These include hsa-miR-943, hsa-miR-935, hsa-miR-367, hsa-miR-363, hsa-miR-25, and hsa-miR-196b. These hub microRNAs are in relation with five hub genes individually. Some of these hub genes show the highest number of commonality between miRNAs, namely SESN3, KCNA4, TESK1, and SOD1. Furthermore, enrichment analysis of the hub genes implies of presences of five important biological processes. Dysregulated miRNAs that regulate these genes could interfere with the normal function of these processes. Text mining of biological processes corresponding to the hub agents could be significant for understanding their roles in H.pylori underlying mechanism. As indicated by GO analysis, “positive regulation of cardiac muscle contraction” is the leading connected process with the hub genes. The involvement of these genes suggests that their expression changes in gastric cancer HP+ could induce some malfunction in cardiac-related processes as well. Previously, it has been reported that there are other diseases associated with the H.pylori infection such as cardiovascular diseases as one of the main reasons of death worldwide (16). Here, it was observed that gastric cancer induced with H.pylori could later develop heart conditions. Hence, there is a possibility that the eradication of H.pylori could reduce both the number of gastric cancer and cardiovascular cases. ADRA1A, KCNA4, SOD1, and SESN3 were found in this study as hub genes related in this group of processes. The next process, “Histone deacetylation” activity for cyclooxygenase 2 has also been indicated previously in gastric cancer (17). In addition, H.pylori showed effective influence on histone changes in gastric cancer as well. Histone H3 at serine 10 (H3 Ser10) and the deacetylation in H3 lysine 23 have been reported (18). HDAC8 is the gene related to this group of processes. 

DNA alkylation group is the third ranked process introduced with TDRKH. In this process, alkylation of DNA results in cell death (19). The accumulation of these events may promote tumorigenesis. In addition, DNA methylation as a part of this group is also an important processes in cancer development (20). H.pylori could causes DNA strand breakage of the infected host cells through alkylation (21, 22). The dysregulation of microRNAs targeting TDRKH could be a part of mechanism through which H.pylori imposes its genome instability. TESK1 plays some role in the next process, positive regulation of actin filament. This process may be implicated in metastasis and invasive development in tumors including gastrointestinal tract (23). H.pylori probably contributes to the progress of the gastric cancer through this process (24). 

One of the altered cellular metabolisms in cancer is lipid metabolism. Lipid storage as a part of this process could be important in gastric cancer induced by H.pylori (25, 26). It could further be associated with cardiovascular diseases and augmentation of its chance. The related gene in this category is PLIN2. It is suggested that H.pylori mainly interferes with these biological processes through down-regulation of the six microRNAs, hsa-miR-943, hsa-miR-935, hsa-miR-367, hsa-miR-363, hsa-miR-25, and hsa-miR-196b targeting linked genes. Malfunctions of these processes were introduced for H.pylori pathogenicity as indicated above. Thus, these dysregulated microRNAs could be responsible for development of gastric cancer and other diseases including heart disease induced by H.pylori. Targeting these regulators could be beneficial for treatment purposes. It can be concluded that the candidate hub microRNAs regulators may provide some clues in the role of H.pylori in gastric cancer and may ultimately serve as candidate biomarkers.

## Conflict of interests

The authors declare that they have no conflict of interest.
